# Consistent Paternity Skew through Ontogeny in Peron's Tree Frog (*Litoria peronii*)

**DOI:** 10.1371/journal.pone.0008252

**Published:** 2009-12-14

**Authors:** Craig D. H. Sherman, Erik Wapstra, Mats Olsson

**Affiliations:** 1 School of Life and Environmental Sciences, Deakin University, Waurn Ponds, Victoria, Australia; 2 School of Zoology, University of Tasmania, Hobart, Tasmania, Australia; 3 School of Biological Sciences, The University of Wollongong, Wollongong, New South Wales, Australia; University of Liverpool, United Kingdom

## Abstract

**Background:**

A large number of studies in postcopulatory sexual selection use paternity success as a proxy for fertilization success. However, selective mortality during embryonic development can lead to skews in paternity in situations of polyandry and sperm competition. Thus, when assessment of paternity fails to incorporate mortality skews during early ontogeny, this may interfere with correct interpretation of results and subsequent evolutionary inference. In a previous series of *in vitro* sperm competition experiments with amphibians (*Litoria peronii*), we showed skewed paternity patterns towards males more genetically similar to the female.

**Methodology/Principal Findings:**

Here we use i*n vitro* fertilizations and sperm competition trials to test if this pattern of paternity of fully developed tadpoles reflects patterns of paternity at fertilization and if paternity skews changes during embryonic development. We show that there is no selective mortality through ontogeny and that patterns of paternity of hatched tadpoles reflects success of competing males in sperm competition at fertilization.

**Conclusions/Significance:**

While this study shows that previous inferences of fertilization success from paternity data are valid for this species, rigorous testing of these assumptions is required to ensure that differential embryonic mortality does not confound estimations of true fertilization success.

## Introduction

Patterns of paternity in offspring are routinely used to infer fertilization success for analysis of mating system evolution, genetic compatibility of mates, or innate competitive ability in sperm competition [e.g. 1], [2], [3]. However, such studies are fraught with potential biases introduced by developmental arrest, selective abortion, or other forms of compromised ontogeny. Few studies have directly tested paternity at fertilization and followed it through various stages of ontogeny [although see 4], [5]. This is particularly problematic in internally fertilizing species where it has been difficult to directly observe developing embryos. Externally fertilizing species are increasing being used in studies of sexual selection as they offer excellent systems for separating out genetic and nongenetic effects on fertilization success and offspring fitness as their external mode of fertilization makes them particularly amenable to controlled laboratory studies using *in vitro* fertilization [e.g.], [2], [6], [7], [8], [9,10]. These systems also allow for the direct assessment of fertilization success and mortality can be followed through various stages of ontogeny, something that is much less tractable in internally fertilizing species.

We show elsewhere that the probability of paternity in the Peron's tree frog (*Litoria peronii*) under conditions of sperm competition is influenced by the relatedness of the two competing males in relation to that of the female [2], and recent data demonstrate that among-male differences in siring success also has an innate component so that male-male differences in siring success are consistent across females [10]. Thus, under conditions of sperm competition the probability of paternity is typically highly skewed towards one of the competing males. However, such effects may not reflect the competitive ability of sperm at fertilization and could potentially be the spurious outcome of selective mortality of developing tadpoles with respect to genetic incompatibility of partners and is predicted to show cumulative severity in proportional mortality through embryonic development. Indeed, a number of recent empirical and theoretical studies caution against the use of paternity data to infer fertilization success in studies of postcopulatory sexual selection [8], [11], [12]. For example, fertilization rates in the sea urchin *Heliocidaris erythrogramma* show no correlation with hatching rates and embryo viability [8], while simulations models of sperm competition trials show that the degree of inequality between paternity and fertilization estimates will depend on the variance in sperm competitiveness, the variance in the ability of males to induce embryo viability and on the relationship between these two traits [11]. Thus differential survival of embryos caused by intrinsic male effects may lead to an overestimation of good gene effects when fertilization success is inferred from paternity analysis.

Hatching success in laboratory studies of amphibians can be highly variable both within and among species. For example, hatching success in the Australian tree frog, Litoria peronii, is typically around 70% but can vary from 0–100% for some individuals (Sherman unpublished data) while Dziminiski et al. [6] reported hatching success of 88%±13% in the polyandrous quacking frog, *Crinia georgiana*. Lower rates of hatching success (7%) have been reported for laboratory studies of the threatened Wyoming toad [13]. Thus it is clear that mortality of developing embryos through ontogeny can be significant in many amphibian species and poses a potential problem for studies that infer fertilization success based on paternity determined at hatching.

In order to test for selective mortality of developing tadpoles through ontogeny, we staged laboratory experiments where paternity of two competing males in a situation of sperm competition was sampled at three time intervals throughout embryonic development. Using nine microsatellite markers to assign paternity, we then tested whether the proportional paternity between the two competing males changed significantly between sampling events throughout embryonic development to test for selective mortality due to inbreeding or other forms of genetic, parental incompatibility.

## Results

### Fertilization and hatching success

The mean fertilization success across all trials was 62%±4% (s.e.m) and we detected no significant differences in fertilization success between replicates within a trial (*F*_2, 45_ = 0.10, *p* = 0.9). Hatching success varied between trials from 46–100% with an average of 82%±3% (s.e.m) of eggs hatching across all 16 sperm competition trials.

### Paternity assessment through ontogeny

**Figure 1:**
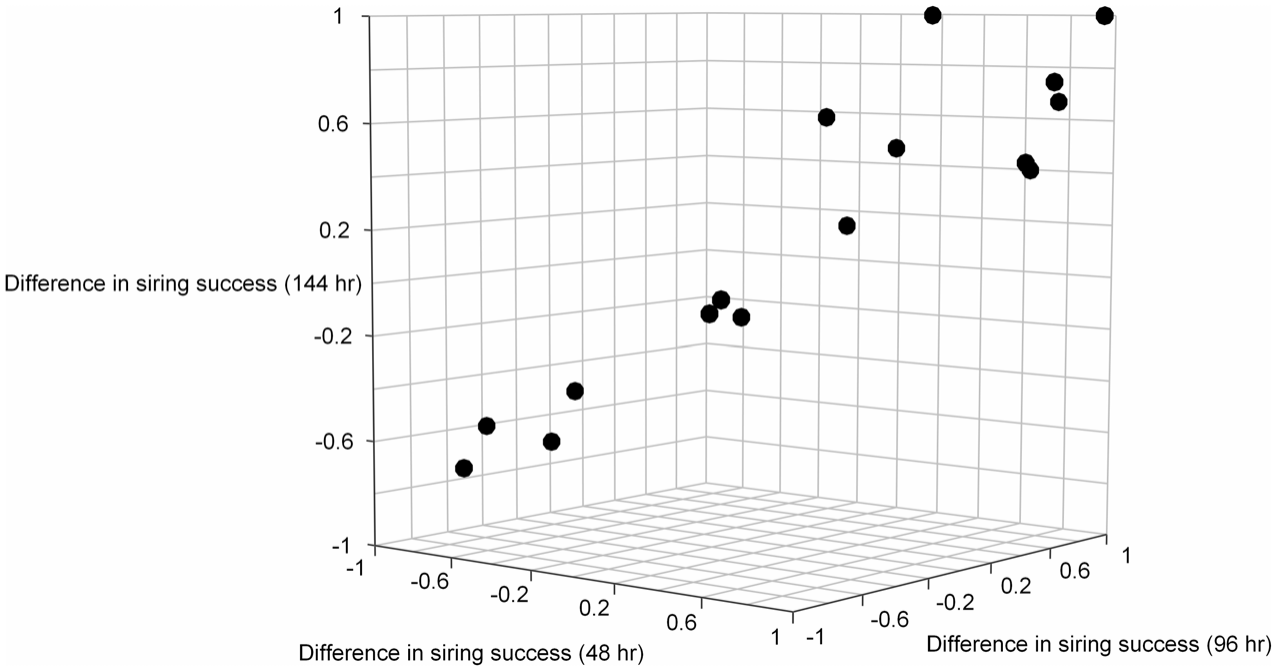
Difference in siring success of competing males in sperm competition determined at three periods during embryonic development.

Based on the nine microsatellite loci we were able to unambiguously assign paternity of each tadpole to one of the two putative sires. Paternity assessed at the first time interval (48 hr post fertilization) was skewed (>70%) towards one of the competing males in 11 of the 16 sperm competition trials. For the remaining two time intervals, 10 of the 16 sperm competition trials remained highly skewed. The two-way mixed intraclass correlation coefficient was highly significant (*ρ* = 0.97, *F*_15, 30_ = 29.9, *P*<0.001) indicating that that the share of paternity of males at different developmental stages are highly correlated (Figure 1). A Repeated Measures ANOVA confirmed that there was no significant change in the share of paternity of competing males during embryonic development (*F*_2, 30_ = 0.01, *P* = 0.99).

## Discussion

Despite recent cautioning on the use of paternity data to infer fertilization success in situations of sperm competition, our results show robust and conclusive support for the hypothesis that biased paternity success sampled late in the embryonic development of the Peron's tree frog is consistent with the corresponding between-male difference in siring success at fertilization. While some clutches can show significant post-fertilization mortality, this does not appear to result from incompatibility effects between maternal and paternal genomes. Thus, in this species there appears to be no mortality skews taking effect in a progressive manner through ontogeny as would be expected if parental genetic incompatibility affects offspring probability of survival during that period of development, as demonstrated in mice [14] and some insects [e.g., 15].

Species with internal fertilization may have a greater opportunity to influence a male's share of paternity compared with externally fertilizing species. This is because there is greater opportunity for cryptic female choice through sperm selection within the reproductive tract, selective abortion or through genetic incompatibility effects [16]. Nevertheless, genetic incompatibility effects on the probability of offspring survival have been shown for some externally fertilizing species [e.g. 8] and we cannot discount the role of biased mortality of developing embryos in other situations such as when levels of inbreeding are higher, or environmental conditions provides stronger selection for offspring with particular genotypes. Further work is required to fully understand when skewed mortality does occur [e.g.], [14], [15,17] and when it does not (our study). Indeed, recent crosses between this species and the closely related *L. tyleri* show no pre-zygotic barriers to fertilization, but severe post-fertilization genetic compatibilities of hybrid offspring [18]. Thus, fertilization success in competitive fertilization trials inferred from later stage offspring would erroneously suggest pre-zygotic barriers which clearly do not exist in these closely related species. Clearly, studies of sperm competition need to rigorously validate the use of paternity data to infer fertilization success and distinguish between pre- and post-fertilization processes that determine a male's ultimate share of paternity. While acknowledging that for species with internal fertilization and development, logistic constraints may prevent the sampling of embryos directly after fertilization, ideally embryos should be sampled as soon after fertilization and before embryo mortality occurs.

## Materials and Methods

### *In vitro* fertilisations and sperm competition trials

The Peron's tree frog, *L. peronii*, is of medium size (up to 6 cm) and common to New South Wales, southern Queensland, eastern and central Victoria and South Australia along the Murray drainage system [19]. They have a prolonged summer breeding season that lasts from late September through to February. The importance of polyandry in this species has not been confirmed by direct analysis of multiple paternity in clutches, however, the observed mating behavour of adults strongly suggests that polyandry is likely to play a role in the mating system of this species. The operational sex ratio is highly skewed towards males and there is intense competition (often involving physical wrestling) among males for access to females. Multiple males have been observed trying to amplex single females and amplexing pairs are often surrounded by satellite males as they move into the water to spawn [11]. We collected adult male and female frogs (*Litoria peronii*) for *in vitro* sperm competition trials from a pond at Darkes Forest, NSW, Australia. Females were caught as they moved down to the breeding pond to spawn. Thus females were naturally primed for egg release and no hormonal priming was necessary to induce ovulation. A total of 16 sperm competition trials were carried out using ejaculates from two randomly chosen males with a randomly chosen female. We induced males to release sperm after a subcutaneous injection of luteinizing hormone releasing hormone (approximately 150 µl per 10 g body weight of a 5 mg/100 ml in isotonic saline solution). This induces males to shed their sperm within 1 h of the injection. Sperm were released into conical tubes and collected from its apex by gently flushing the conical tube with 3 ml of aged water. Males were injected within two minutes of each other to ensure that longevity of sperm did not confound our experiments. Sperm concentration for each male was determined using a Hawksley haemocytometer and the sperm samples diluted to equal concentrations (6×10^5^ sperm cells per ml). Three milliliters of the males' sperm solutions were mixed together by pipetting and divided between three replicate Petri dishes. Approximately equal numbers of eggs (112±4.5 s.d) were introduced into each Petri dish from a randomly chosen female by gently squeezing her abdomen directly into the Petri dishes. All females were used within 12 h of capture. After two minutes, the egg/sperm mixture was flooded with 100 ml of aged water. After three hours the eggs in each replicate Pteri dish were transferred to separate 750 ml plastic containers and held at a constant temperature of 23°C. The three replicates from each sperm competition trial were randomly assigned to one of three sampling periods, 48 hours (stage 14–15) 96 hours (stage 18–19) and 144 hours (stage 22–23, hatching) post fertilization [20]. All work was carried out in accordance with National Parks and Wildlife Services permit S11186 and The Wollongong University animal ethics permits AE04/03-05.

### Fertilization and hatching success

Using a dissecting microscope the number of eggs successfully fertilized for each replicate was assessed 48 h post fertilization when developing embryos could be distinguished from unfertilized eggs. Hatching success was determined as the number of tadpoles that successfully hatch at 144 hours.

### Collection of embryos and assignment of paternity

From each sperm competition trial we sampled 25 embryos at 48 hours post fertilization. A further 25 embryos were then collected from each sperm competition trial at 96 hours and 144 hours post fertilization. A toe clip from each adult was used for DNA extraction and the assignment of paternity. Genomic DNA was isolated using Qiagen DNAeasy Tissue Kit as per the manufacturer's instructions. Nine microsatellite loci (LP01, LP03, LP04, LP05, LP07, LP13, LP19, LP22 and LP23) were used to assign paternity [21]. Paternity was unambiguously assigned to all offspring according to allele sharing between putative sires, dam and offspring.

### Statistical analysis

We used a one-way ANOVA to test for differences in fertilization success (assessed at 48 hours) between replicates within sperm competition trials. The data met the assumption of normality and homogeneity of variance. To determine if the share of paternity of competing males in sperm competition changed during embryonic development, we calculated a reliability analysis using the two-way mixed intraclass corrrelation coefficient option in SPSS (Version 16). The intraclass correlation coefficient measures the correlation or consistency of a data set with multiple groups [22]. Thus, the intraclass correlation coefficient, Rho (*ρ*), tests for consistency in the share of paternity across the three different developmental periods sampled.

We then tested for significant effects of embryonic development period on the share of paternity using a Repeated Measures two-way ANOVA (PROC MIXED, SAS 9.1) with the difference in the proportion of offspring sired between competing males as the response variable and time and female as the two predictors in the model. The difference in the proportion of offspring sired by competing males in sperm competition met the assumptions of normality and homogeneity of variances.
